# Algorithmic fairness audits in intensive care medicine: artificial intelligence for all?

**DOI:** 10.1186/s13054-022-04197-5

**Published:** 2022-10-18

**Authors:** Davy van de Sande, Jasper van Bommel, Eline Fung Fen Chung, Diederik Gommers, Michel E. van Genderen

**Affiliations:** Department of Adult Intensive Care, Erasmus University Medical Center, Room Ne-403, Doctor Molewaterplein 40, 3015 GD Rotterdam, The Netherlands

**Keywords:** Artificial intelligence, Intensive care, Bias, Equity

Research on artificial intelligence (AI) has emerged as a promising field that has the potential to improve patient outcomes, for example, by optimizing timing of antibiotic therapy in the intensive care unit (ICU) or by AI-based delirium management, as recently published in this journal [1, 2]. Despite its potential, we have to be aware that not all patients may equally benefit from such advancements; ‘unfair’ or ‘unequal’ AI algorithms could reinforce systemic health disparities. For example, a recent study demonstrated consistent underdiagnosed chest X-ray pathologies by an AI algorithm in black and female patients [3]. In fact, even well-established ICU prediction models could be unfair. During the COVID-19 pandemic, Sequential Organ Failure Assessment (SOFA)-based allocation of ICU resources was proven to have racial inequality and could have induced disparities [4]. These results stress that especially future AI-based ICU interventions, or policies, should be fair and have a similar impact on all patients involved, irrespective of gender, ethnicity, and other protected personal characteristics as recently stated by the World Health Organization (WHO) [5].

One of the reasons AI research has skyrocketed in intensive care medicine [6] is the availability of large publicly available datasets, such as the Medical Information Mart for Intensive Care (MIMIC) [7]. These data are often collected at single site and as such could underrepresent different subpopulations across different ICUs [8]. To illustrate, less than 10% (number: 18,719/189,415) of the patients registered in the two largest ICU databases worldwide are African-American, while the vast majority are white male patients [8]. Given the serious consequences of unequal algorithms that could arise from such biased data [9], and the fact that several methods exist to mitigate such biases [10], it seems clear that an ‘algorithmic fairness audit’ should be part of the development and implementation process. Such an audit should facilitate the evaluation and reporting of an AI algorithms’ performance on specific subpopulations instead of only on the total population, which is the current standard (Fig. 1).

**Fig. 1:**
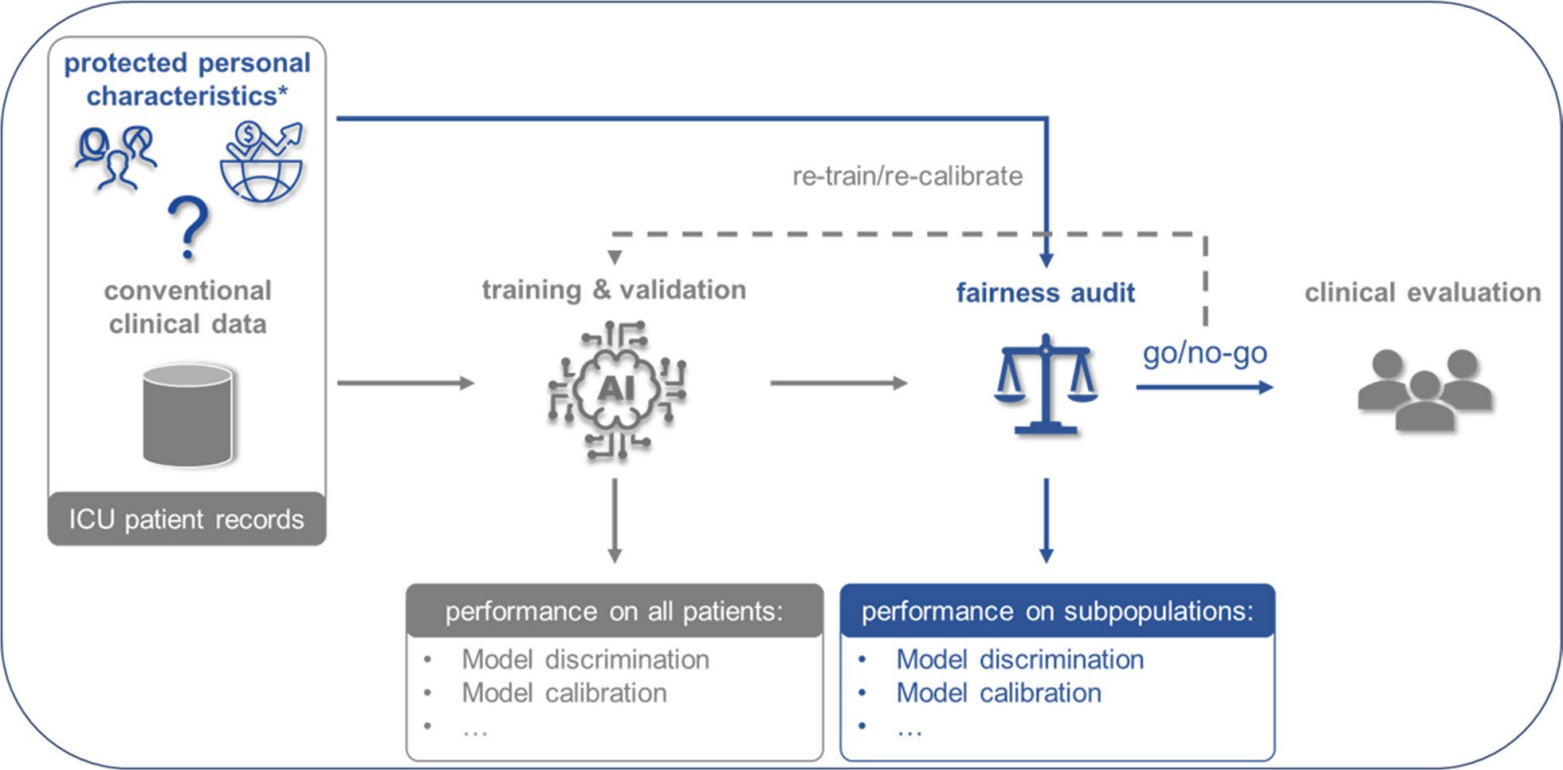
Schematic overview of the intensive care medicine artificial intelligence fairness audit. Conventional clinical patient data (e.g., vital signs, laboratory values, and demographics) are typically used to train an AI algorithm and its performance is then evaluated on an internal or external test dataset to see whether it works in the first place. Next, the fairness audit should take place: evaluate model performance across multiple subpopulations (for example, based on ethnicity, age, gender, or other characteristics). If concerns regarding algorithmic fairness arise, re-training and/or re-calibration should be considered (go/no-go). *Protected personal characteristics such as ethnicity, socioeconomic information, and others need to be collected in patient health records. AI = artificial intelligence

Although we acknowledge the complexity of algorithmic fairness, several practical steps could help to prevent unequal algorithms making their way to ICU patients’ bedside. We therefore outline a couple of them. Firstly, a common understanding of protected personal characteristics (e.g., age, gender, and ethnicity) that, at minimum, should be obtained is crucial to adequately design and perform fairness audits. The real question here is: To which protected personal characteristics should an AI algorithm definitely be fair? In answering this question, we must obviously account for historical (racial) and societal disparities [11] and intensify dialogue between key stakeholders (data protection authorities, editorial teams, patients, ICU professionals, and ethical review boards). In addition, it is known that there may exist ethnical differences in disease manifestation and comorbidity; for example, multimorbidity is more common among African-American patients than white patients [12]. With the above in mind, a list of protected personal characteristics should be composed to uniformly perform and report fairness audits.

Secondly, and based on the former, relevant protected personal characteristics need to be routinely and uniformly collected in patient health records, worldwide. For example, ethnicity and socioeconomic information are typically protected under human rights codes but are unavailable in most ICUs outside of the USA, while age and gender are widely available [8]. In practical terms, this means we have to define specific subpopulations (e.g., define ethnic groups), train healthcare professionals, standardize data collections, and potentially adjust local policies, among others. Several recommendations could already help to collect such information [13], such as implement standardized collection forms in regular health checkups within primary care, link data from primary and secondary care, implement strict terms for use of such data, and periodically evaluate data quality and completeness. Also, several lessons can be learned from existing examples such as the UK, where ethnicity data are already routinely recorded in patient health records.

Lastly, we need to determine which metrics should be used to assess fairness; are standard AI performance metrics (discrimination and calibration) sufficient or do we need fairness-specific metrics? There is a wealth of metrics that can particularly be used to assess whether treatments or predictions are equally divided over individuals or protected patient groups on multiple levels (e.g., are true positives and false positives equally distributed over protected and unprotected groups?, is the false negative and false positive ratio the same between protected and unprotected groups?, or do patients from protected and unprotected groups with the same risk prediction have the same probability of correctly belonging to the positive class?) [10]. The most appropriate metric to choose mainly depends on the context of the clinical problem; there is no one size that fits all [14]. As a starting point, an AI algorithms’ discrimination and calibration should be evaluated on various subpopulations before making the step toward clinical implementation. Also, depending on the context additional fairness-specific metrics should be determined.

To improve algorithmic fairness, we therefore advocate for a standard fairness audit based on readily available data (age and gender), when developing and implementing AI algorithms in the ICU. Parallel to this, protected personal characteristics should be identified and collected to thoroughly evaluate fairness outcomes on multiple aspects in the future. Also, as the maturity of AI in intensive care medicine is expected to shift in the upcoming years from development to clinical implementation, (unforeseen) ethical considerations become increasingly important [15]. An AI fairness audit should be part of a larger set of ethical considerations to warrant safe and fair usage of AI in the ICU field. We are currently composing such a set based on the WHO guidance on AI ethics [5] (PROSPERO database ID: CRD42022347871).

## Data Availability

Not applicable.

## Abbreviations

AI: Artificial intelligence
ICU: Intensive care unit
MIMIC: Medical Information Mart for Intensive Care
UK: United Kingdom
WHO: World Health Organization
